# Patterns of condom use by men who have sex with men before and after the Avahan intervention in Andhra Pradesh state of India

**DOI:** 10.1186/1471-2458-14-64

**Published:** 2014-01-22

**Authors:** G Anil Kumar, Rakhi Dandona, Ramesh Poluru, S Anil Chandran, Michel Alary, Lalit Dandona

**Affiliations:** 1 Public Health Foundation of India, New Delhi, India; 2 Indian Institute of Public Health, Hyderabad, India; 3 Département de médecine sociale et préventive, URESP, Centre de recherche du CHU de Québec, Université Laval, Québec, Canada; 4 Institute for Health Metrics and Evaluation, University of Washington, Seattle, WA, USA

**Keywords:** Condom use, HIV, India, Men who have sex with men, Regular sex partner

## Abstract

**Background:**

Two rounds of integrated biological and behavioural assessment (IBBA) surveys were done among men who have sex with men (MSM) in Andhra Pradesh during 2006 and 2009. Avahan, the India AIDS initiative, funded by the Bill and Melinda Gates Foundation implemented HIV prevention interventions among MSM starting around the time of the first round of IBBA.

**Methods:**

Data on socio-demographic, sex behaviour characteristics and HIV status of MSM from the two IBBA rounds were used. Changes in the rates of consistent condom use over the past one month by MSM with various types of partners between the two rounds were assessed. Multivariate analysis was performed to assess associations between various factors and inconsistent condom use for sex with regular partners as well as HIV in MSM.

**Results:**

A significant increase in consistent condom use by MSM was noted from 2006 to 2009 for paid male partners (19.5% to 93.8%), occasional male partners (13.2% to 86.2%), and paid female partners (25.9% to 94.2%). Consistent condom use with regular sex partners also increased but remained lower with regular male partner (75.8%) and very low with regular female partners (15.7%). MSM who used condoms inconsistently with their regular male partner were also more likely to use condoms inconsistently with their regular female partner. Multivariate analysis showed MSM who used condoms inconsistently with regular male partner had higher odds of HIV (odds ratio 1.8; 95% CI 1.2-2.7). MSM who received condoms from Avahan had the lowest odds (odds ratio 0.3; 95% CI 0.1-0.5) of inconsistent condom use with regular male partners.

**Conclusions:**

Condom use by MSM increased markedly after implementation of Avahan, though a causal association cannot be assessed with the available data. The relatively lower condom use with regular partners of MSM suggests that additional programme effort is needed to address this aspect specifically.

## Background

HIV in men who have sex with men (MSM) influences the overall HIV epidemic in both low and high income countries, which is related to high per-act and per-partner transmission probability of HIV transmission in receptive anal sex [1-4]. According to the sentinel surveillance of the National AIDS Control Organization of India, HIV prevalence in MSM in 2008–2009 was estimated to be 7%, which is about 20 times higher than the overall national adult HIV prevalence rate [5].

Various MSM programmes were started in India in the last decade to reduce HIV including in Andhra Pradesh which is one of the high HIV prevalence states in India [6,7]. Avahan, the India AIDS Initiative, funded by the Bill and Melinda Gates Foundation was one among these [8]. The Avahan MSM programmes in Andhra Pradesh started between October 2005 and April 2006 [9]. Avahan intervention was conceived as a focused prevention program, offering a standardized package of proven prevention interventions for high-risk groups, such as community participation, clinical services for STI and counselling, peer lead outreach and commodity distribution [8]. Two rounds of integrated biological and behavioural assessment (IBBA) surveys were conducted among MSM in Andhra Pradesh in April-June 2006 and April-June 2009 to assess the effect of intervention programme [9]. The first IBBA round was within six months of start of the Avahan MSM programmes, and therefore can be considered as baseline for this intervention.

Previous studies have suggested that many MSM in India have a mix of paid, occasional and regular male and female partners [10-15]. Consistent condom use by MSM in Andhra Pradesh has been previously reported to be low with both male and female partners [11,14,16], though there has been a suggestion of increase in condom use with paid partners [17]. In order to understand how the MSM intervention programmes can be improved further, this paper reports the changes in consistent condom use by MSM with various types of partners from 2006 to 2009 in Andhra Pradesh from the two IBBA rounds, and the associations between various factors and inconsistent condom use for sex with regular partners as well as HIV in MSM.

## Methods

Multiple rounds of IBBA surveys were designed to evaluate the impact of Avahan programme on populations at high risk as well as provide data on changes in risk behaviours and prevalence of STI including HIV. Both rounds of IBBA covered different categories of high risk population, including MSM, female sex workers and their clients. The operational definition used for MSM in both surveys was “any male or *hijra* (eunuch) aged 18 years or older who had any type of sex with another male in the last one month” [9,18].

### Survey procedures

Two independent cross-sectional IBBA surveys were implemented in Andhra Pradesh as part of an evaluation of the Avahan initiative among high risk group [9,18]. These surveys were carried out among MSM in four districts in Andhra Pradesh, namely East Godavari, Guntur, Hyderabad and Visakhapatnam. The IBBA survey in Andhra Pradesh was led by the National Institute of Nutrition, and the field work was done by a hired research agency AC Nielsen ORG MARG Private Ltd for both rounds under the guidance of the National Institute of Nutrition. The National AIDS Research Institute coordinated the conduct of the IBBA survey at the national level and FHI 360 provided technical assistance for implementing the IBBA. The details of study design are available elsewhere [9,18].

Relevant for this report, training was given to the survey team about survey protocol, questionnaire administration, sample collection and transport of biological samples. Written informed consent was obtained from all participants. A sample size of 400 self-identified MSM per district was interviewed face-to-face and biological specimen collected with a two-stage cluster sampling design. Fixed-location and time-location clusters were the sampling strategy in East Godavari district. In the other three districts, only time-location clusters were considered and recruitment of high-risk MSM for the survey was predominantly from public places. Data were collected from a total of 1,621 and 1,608 sampled self-reported MSM from Andhra Pradesh in round 1 and round 2 of IBBA surveys, respectively. Both rounds of IBBAs collected detailed behavioural information including socio-demographic characteristics, sexual history, practices, type of sex partners, condom use, exposure to Avahan or other HIV/AIDS prevention initiatives, and biological specimens to test for HIV and STIs. Standardized laboratory methods were used for HIV testing. Double-data entry of district-level datasets was conducted using CSPro software (U.S. Census Bureau, Washington DC) for both rounds of IBBA. Centralized data management was done for IBBA round one by the National Institute for Epidemiology, Chennai and decentralized data management was done in round two in each state.

The protocol for the IBBA surveys was approved by the Health Ministry Screening Committee and the Indian Council of Medical Research, and ethics approval was given by the FHI Protection of Human Subjects Committee [19].

### Statistical analysis

We used data on condom use by MSM with different partners in each round of IBBA survey to measure the change in consistent condom use between 2006 and 2009. Data are presented for regular partners and partners who were paid for sex. Data are not presented for partners who paid for sex because the variable used for this in the two IBBA rounds was different which did not allow direct comparison. We defined consistent condom use if condom was used every time during sex over the past one month with different sex partners. The choice of past one month was based on the availability of this variable in both IBBA rounds for all types of partners that we considered, whereas condom use data over longer duration was not available in the first round of IBBA for partners who were paid. Appropriate sample weights were used for estimating consistent condom use and HIV prevalence among MSM; the 95% confidence intervals were calculated taking into account the design effect of the cluster sampling strategy [20].

A detailed analysis of the variables associated with inconsistent condom use and with HIV was performed for the second round of IBBA data using multivariate analysis. We used multiple logistic regression models to assess the association of socio-demographic variables and behavioural variables with inconsistent condom use by MSM with regular male and female sex partners. We included these variables in the models: age, education, marital status, ever received cash or kind for sex, paid to have anal intercourse with a male or *hijra* ever, circumcision, duration of sex with male, received condoms from the peers or outreach workers of the NGO/programme, and membership in community-based organization. We used a similar approach for assessing the associations with HIV in MSM. In addition to the previously mentioned variables, we also assessed the association of inconsistent condom use with regular male partner with HIV.

A total of 13 respondents, 5 in IBBA round 1 and 8 in round 2, did not report any male sex partner, and were therefore not considered for the analysis of MSM in this paper. Analysis was done using SPSS 17.0 (IBM SPSS statistics standard, USA) and STATA 11.2 (StataCorp, USA) software.

## Results

### Sample characteristics

The distribution of the demographic and sex behaviour characteristics of MSM in the two rounds of IBBA surveys are presented in Table 1. The proportion of literate and never married MSM was slightly higher in the second IBBA round. As compared with the first round, in the second round a lower proportion of MSM reported paid male sex partners while a slightly higher proportion reported occasional male sex partners, and a substantially lower proportion reported a paid female sex partner. The proportion of MSM who reported membership in community based organization jumped from 2.9% in first IBBA round to 65.8% in the second round, as might be expected after the Avahan initiation.

**Table 1:** Characteristics of MSM in the two IBBA rounds

**Variable**	**Categories**	**Number (% of N) [95% CI of % or years]**	
		**IBBA Round 1**	**IBBA Round 2**
		**2006**	**2009**
		(N = 1,618)*	**(N = 1,600)†**
Age (years)	Mean ± SD	27.5 ± 8.0	27.3 ±7.4
		[27.0 - 28.0]	[26.5 - 28.0]
Education	Illiterate	381 (23.5)	288 (18.0)
		[21.5 - 25.6]	[16.1 - 19.9]
	Literate	1,237 (76.5)	1,312 (82.0)
		[74.4 - 78.5]	[80.1 - 83.9]
Marital status with female	Never married	900 (55.6)	1,015 (63.4)
		[53.2 - 58.0]	[61.0 - 65.8]
	Ever married	719 (44.4)	586 (36.6)
		[42.0 - 46.8]	[34.2 - 39.0]
Circumcised	No	1,435 (88.9)	1,471 (91.9)
		[87.3 - 90.4]	[90.6 - 93.3]
	Yes	179 (11.1)	129 (8.1)
		[9.6 - 12.7]	[6.7 - 9.4]
Male sex work	No	859 (53.1)	878 (54.9)
		[50.7 - 55.6]	[52.4 -57.3]
	Yes	758 (46.9)	722 (45.1)
		[44.4 - 49.3]	[42.7 - 47.6]
Male Partners‡	Regular	1,032 (63.8)	979 (61.2)
		[61.5 - 66.1]	[58.8 - 63.6]
	Paid	696 (43.0)	328 (20.5)
		[40.6 - 45.4]	[18.5 - 22.5]
	Occasional	1,460 (90.2)	1,522 (95.1)
		[88.8 - 91.6]	[94.0 - 96.2]
Female partners‡	Regular	823 (50.9)	706 (44.1)
		[48.5 - 53.3]	[41.7 - 46.5]
	Paid	656 (40.6)	99 (6.2)
		[38.2 - 43.0]	[5.0 - 7.4]
Age at first sex with a man (years)	Mean ± SD	16.4 ± 2.5	17.1 ±2.7
		[16.3 - 16.6]	[16.9 - 17.4]
Duration of sex with men (years)	Mean ± SD	11.1 ± 7.7	10.1 ±7.5
		[10.6 - 11.6]	[9.4 - 10.9]
Membership in MSM community-based organization	No	1,571 (97.1)	552 (34.7)
		[96.2 - 97.9]	[32.3 - 37.0]
	Yes	47 (2.9)	1,040 (65.3)
		[2.1 - 3.8]	[63.0 - 67.7]
HIV status	Negative	1,338 (82.7)	1,296 (81.0)
		[80.8 - 84.5]	[79.1 - 82.9]
	Positive	280 (17.3)	304 (19.0)
		[15.5 - 19.2]	[17.1 - 20.9]

*Data missing in IBBA Round 1: circumcision for 4; male sex work for 1; duration of sex with male for 2.

†Data missing in IBBA Round 2: age for 1; duration of sex with male for 1; paid male partner for 10; membership in a community based organization for 9.

‡Not mutually exclusive.

### Consistent condom use with different sex partners

**Figure 1:**
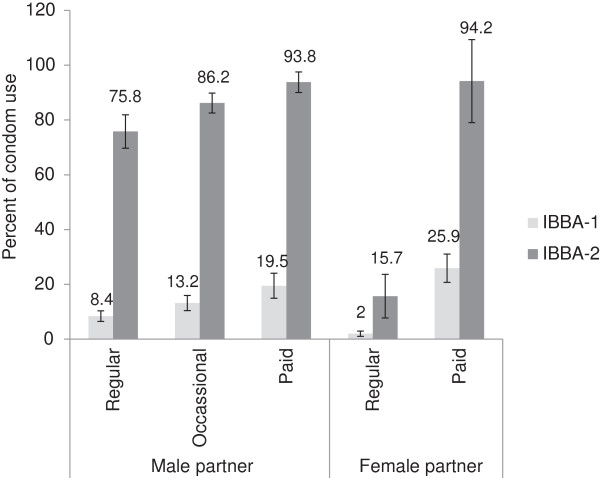
**Consistent condom use every time in the last one month by MSM with different sex partners in the two IBBA rounds.** The truncated bars indicate 95% confidence intervals. Regular male sexual partner means lover/boyfriend. Occasional male partner means non-regular and non-paid male sex partner. Paid male partner means paid to have anal sex with male/*hijra.* Regular female sexual partner means spouse/lover/girlfriend. Paid female partnr means paid to have sex with female.

The proportion of MSM who reported consistent condom use for each sex episode over the past one month with paid male partners improved considerably from 19.5% (95% CI 14.9-24.1, design effect 2.1) in IBBA round 1 to 93.8% (95% CI 90.0–97.6, design effect 11.6) in IBBA round 2, and a similar trend was seen for occasional male partners as well (Figure 1). Even though there was a substantial increase in consistent condom use with regular male partners from 8.4% (95% CI 6.5-10.3, design effect 1.3) in round 1 to 75.8% (95% CI 69.7-81.9, design effect 4.9) in round 2, a quarter of the MSM were still not using condoms consistently with their regular male partners. A significant increase was also seen in consistent condom use by MSM with female paid partners from 25.9% (95% CI 20.7-31.1, design effect 2.1) in round 1 to 94.5% (95% CI 79.1-100, design effect 10.6) in round 2. However, only 15.7% (95% CI 7.8-23.6, design effect 8.7) MSM reported using condoms consistently over the past one month with their regular female partner in IBBA round 2. Among the 223 MSM who reported inconsistent condom use with their regular male partner, 81 (36.2%) also had a regular female partner of whom 78 (96.9%) did not use condoms consistently with her. On the other hand, among the 699 MSM who reported consistent condom use with their regular male partner, 272 (39%) also had a regular female partner of whom 211 (77.5%) did not use condoms consistently with her.

It is interesting to note that in the IBBA round 2, of the 702 MSM who used condom consistently with their regular male partner over the last one month, 681 (97%) also reported using condom consistently with their regular male partner over the last six months, indicating that these proportions were not very different between one and six months. Similarly, of the 111 MSM who used condom consistently with their regular female partner over the last one month, 100 (90.1%) also reported using condom consistently with their regular female partner over the last six months.

### Associations with inconsistent condom use with regular male partner

Of the 1,600 MSM in IBBA round 2, 979 (61.2%) reported having had a regular male sex partner. Among them 926 (94.5%) ever had anal sex with him, of which 223 (24.2%) did not use condoms for over the past one month. Table 2 shows the variables associated with inconsistent use of condom by MSM with their regular male partners. MSM who received condoms from peers or outreach workers of Avahan NGO had lower odds of inconsistent condom use with their regular male partners (odds ratio 0.3; 95% CI 0.1-0.5). The following MSM were more likely to use condoms inconsistently with their regular male partners: those who never received cash or kind for sex (odds ratio 2.4; 95% CI 1.6-3.5), those with 6 or less years of duration of sex with males (odds ratio 2.2; 95% CI 1.3-3.6), and those aged 25–34 years (odds ratio 1.9; 95% CI 1.2-3.1).

**Table 2:** **Association of socio**-**demographic and behavioural variables with inconsistent condom use by MSM over the past one month with their regular male and female sex partners**, **using multiple logistic regression**

**Variable**	**Categories**	Total* (N = 926)	**Inconsistent condom use with regular male partner**		**Total† (N = 706)**	**Inconsistent condom use with regular female partner**	
			**Number (%)**	**Odds ratio (95% CI)**		**Number (%)**	**Odds ratio (95% CI)**
Age (years)	18-24	402	96 (23.9)	1.0	147	62 (42.28)	1.0
	25-34	388	99 (25.5)	1.9 (1.2 -3.1)	356	337 (94.7)	4.8 (1.3-17.2)
	>34	133	29 (21.8)	1.7 (0.8 -3.4)	200	194 (97.0)	2.0 (0.4-9.6)
Education	Illiterate	161	37 (23.0)	1.1 (0.7 -1.7)	137	133 (97.1)	4.7 (1.2-18.0)
	Literate	762	187 (24.5)	1.0	568	461 (81.2)	1.0
Marital status with female	Ever married	281	71 (25.3)	1.3 (0.8-2.1)	568	556 (97.9)	51.6 (18.4-144.5)
	Never married	641	152 (23.7)	1.0	136	37 (27.2)	1.0
Ever received cash or kind for sex	No	453	134 (29.6)	2.4 (1.6-3.5)	443	404 (91.2)	5.0 (2.3-11.1)
	Yes	469	89 (19.0)	1.0	261	190 (72.8)	1.0
Paid to have anal intercourse with a male or *hijra* ever	No	748	196 (26.2)	1.4 (0.9-2.4)	577	506 (87.7)	1.5 (0.7-3.3)
	Yes	173	27 (15.6)	1.0	128	88 (68.8)	1.0
Circumcision	No	844	195 (23.1)	1.0	628	524 (83.4)	1.3 (0.3-5.0)
	Yes	78	28 (5.9)	1.5 (0.9-2.7)	77	70 (90.9)	1.0
Duration of sex with male (years)	<=6	292	90 (30.8)	2.2 (1.3-3.6)	148	73 (49.3)	1.0
	> 6	630	133 (21.1)	1.0	556	521 (93.7)	1.5 (0.4-5.4)
Received condoms from the peers or outreach workers of the NGO/programme in past 1 year	None	146	65 (44.5)	1.0	180	122 (66.7)	1.0
	Avahan	551	72 (13.1)	0.3 (0.1-0.5)	424	380 (89.6)	0.5 (0.2-1.1)
	Non-Avahan	216	85 (39.4)	1.8 (0.9-3.6)	97	92 (94.8)	1.2 (0.3-5.3)
Membership in MSM community-based organization	No	229	84 (36.7)	1.3 (0.7-2.4)	243	160 (65.8)	1.0
	Yes	689	139 (20.2)	1.0	459	432 (94.1)	3.8 (1.5-9.6)

*MSM who had regular male partner and had anal sex with him.

†MSM who have regular female partner and had sex with her.

### Associations with inconsistent condom use with regular female partner

Of the 1,600 MSM in IBBA round 2, 706 (44.1%) reported having had a regular female sex partner and ever had sex with her. Of these, 84% did not use condom for each sex episode over the past one month with their regular female partner. Table 2 shows the association of inconsistent condom use by MSM with their regular female partner over the past one month. The strongest variable associated with inconsistent condom use was MSM who were ever married with female (odds ratio 51.6; 95% CI 18.4-144.5). The other significant associations with inconsistent condom use were with never received cash or kind for sex (odds ratio 5.0; 95% CI 2.3-11.1), age 25–34 years (odds ratio 4.8; 95% CI 1.3-17.2), and membership in an MSM community-based organization (odds ratio 3.8; 95% CI 1.5-9.6). In contrast, a higher proportion of MSM who had membership in an MSM community-based organisation used condoms consistently with their non-regular male partners and regular male partner as compared with other MSM (85.6% versus 77% [p = 0.034] and 79.8% versus 63.3% [p < 0.001], respectively). Of the 1,040 MSM who reported membership in a community-based organization, 440 (42.3%) reported being ever married, whereas among the other 552 MSM only 144 (26.1%) reported being ever married.

### Associations with HIV

The HIV sero-prevalence among MSM reported in the two IBBA rounds was not significantly different: IBBA round 1 (17.3%, 95% CI 14.1-20.5; design effect 3.1) and IBBA round 2 (19%, 95% CI 15.4-22.6; design effect 3.4). The district-wise HIV prevalence reported in IBBA round 2 ranged from 4.9% in Visakhapatnam to 28.9% in Hyderabad. Table 3 shows that the MSM who used condoms inconsistently with regular male sex partner had higher odds of HIV (odds ratio 1.8; 95% CI 1.2-2.7). The other significant associations for being HIV positive were more than 6 years duration of sex with male (odds ratio 2.6; 95% CI 1.8-3.9), received condoms from peers or outreach workers of the Non-Avahan NGO/programme (odds ratio 2.1; 95% CI 1.2-3.9), never married MSM (odds ratio 1.7; 95% CI 1.2-2.4), and illiterate MSM (odds ratio 1.4; 95% CI 1.0 -1.9).

**Table 3:** **Association of socio**-**demographic and behavioural variables with HIV**, **using multiple logistic regression**

**Variable**	**Categories**	**Total**	**Being HIV positive**	
		**(N = 1,600)**	**Number (%)**	**Odds ratio (95% CI)**
Age (years)	18-24	676	99 (14.6)	1.0
	25-34	673	146 (21.7)	1.2 (0.8-1.7)
	>34	251	59 (23.5)	1.3 (0.8-2.2)
Education	Illiterate	288	63 (21.9)	1.4 (1.0-1.9)
	Literate	1,312	241 (18.4)	1.0
Marital status with female	Ever married	585	117 (20.0)	1.0
	Never married	1,015	187 (18.4)	1.7 (1.2-2.4)
Ever received cash or kind for sex	No	878	148 (16.9)	1.0
	Yes	722	156 (21.6)	1.1 (0.8-1.4)
Paid to have anal intercourse with a male or *hijra* ever	No	1,262	262 (20.8)	1.4 (0.9-2.1)
	Yes	328	42 (12.8)	1.0
Circumcision	No	1,471	283 (19.2)	1.3 (0.8-2.2)
	Yes	129	21 (16.3)	1.0
Duration of sex with male (years)	<=6	566	62 (11.0)	1.0
	> 6	1,034	242 (23.4)	2.6 (1.8-3.9)
Received condoms from the peers or outreach workers of the NGO/programme	None	426	52 (12.2)	1.0
	Avahan	843	152 (18.0)	1.2 (0.7-2.1)
	Non-Avahan	331	100 (30.2)	2.1 (1.2-3.9)
Membership in MSM community-based organization	No	551	75 (13.6)	1.0
	Yes	1,040	229 (22.0)	1.2 (0.7-1.9)
Inconsistent condom use with regular male partner over the past one month	No	699	103 (14.7)	1.0
	Yes	223	51 (22.9)	1.8 (1.2-2.7)
	No regular partner	678	150 (22.1)	2.3 (1.6-3.1)

## Discussion

This analysis of data from the two rounds of IBBA in Andhra Pradesh reveals that consistent condom use reported by MSM over the past one month increased for all types of sex partners during the three year period from 2006, corresponding to the start of Avahan intervention, to 2009. While the consistent condom use rate by MSM with paid male and female partners reached 94% in 2009, this rate was lower with their regular male partners at 76% and dismally low with their regular female partners at 16%. The MSM who used condoms inconsistently with their regular male partner were also more likely to use condoms inconsistently with their regular female partner. We found a positive association of HIV among MSM with inconsistent condom use with regular male partners. This does not necessarily imply that MSM get HIV from their regular partners, rather that this association likely reflects broader risk exposure of MSM who use condoms inconsistently with regular male partners. While some of the inconsistent condom use by MSM with their regular male partners could be explained by ‘negotiated safety’ between them, generally the inconsistent condom use by MSM with their regular female and male partners needs attention due to their potential to transmit HIV.

Of the MSM in IBBA round 2, 37% were married. Married MSM had very high odds of inconsistent condom use with their regular female partner. Some other studies from India have also reported a very high proportion of unprotected vaginal sex by MSM with their wives [15,17,21-23]. It is plausible that the high level of inconsistent condom use by MSM with their regular female partner, often their wife, is due to concealment of their sex with men from their female partner.

The MSM who received condoms from the Avahan peers or outreach workers had the least odds of inconsistent condom use with their regular male partner. While the much higher proportions of consistent condom use by MSM with their various kinds of partners in 2009 as compared with 2006 is suggestive of a beneficial effect of Avahan in Andhra Pradesh, this cannot be firmly concluded from the data analysed in this paper as there could also have been other contributors to this observed increase. However, two other analyses of the population level effect of Avahan across six states have revealed that Avahan had a beneficial effect in preventing HIV in several states of India including Andhra Pradesh [24,25].

We found a positive association between inconsistent condom use with regular female partner and membership of MSM in community-based organisations. This contrasted with a more consistent condom use by these MSM with their regular and non-regular male partners. While the proportion of ever-married MSM among those with community-based organisation membership was higher than among those without this membership, both this membership and being married had significantly higher odds of inconsistent condom use with their regular female partner in multivariate analysis. This peculiar relation between membership and inconsistent condom use probably reflects some other underlying characteristics of the MSM sample in IBBA second round who had membership of community-based organisations.

The MSM who had never received cash or kind for sex had higher odds of inconsistent condom use with both male and female regular partners. This possibly reflects that MSM who are sex workers are more likely to be exposed to the MSM intervention programmes, indicating that the intervention programmes also need specific efforts to reach MSM who are not sex workers.

MSM who had had sex with males for 6 or less years were less likely to use condoms consistently with their regular male partner, indicating that it would be useful for MSM intervention programmes to try to reach out to early-stage MSMs. On the other hand, MSM who had had sex with males for more 6 years had higher odds of being HIV positive, which likely reflects the cumulative risk of HIV among them and also the possibility that in the past they may not have used condoms as consistently as they were using at the time of the survey.

A significant limitation of this study is that causality between the Avahan intervention and the increase in condom use by MSM observed after its roll-out cannot be ascertained in this pre- and post-intervention study design. In addition, self-reported data about condom use by MSM may be influence by social desirability bias, that is, MSM may have become more aware by the second IBBA round that condom use is expected and therefore they may have over-reported it. Also, there were some socio-demographic differences in the MSM samples of the two IBBA rounds, which may have influenced to some degree the differences observed in the two rounds. On the whole, however, the data presented in this paper indicate the possibility of a beneficial effect of Avahan, which is supported by two other analyses of HIV prevention at the population level that could be attributed to Avahan [24,25].

## Conclusions

Condom use by MSM increased markedly after implementation of Avahan intervention in Andhra Pradesh state in southern India, indicating a beneficial effect, though a causal association cannot be firmly assessed with the available data. However, the continuing very low rate of consistent condom use by MSM with their regular female partners, and to some degree a low rate of consistent condom use with their regular male partners, need particular attention in HIV prevention programmes for MSM in India.

## Competing interests

The authors declare that they have no competing interests.

## Authors’ contribution

LD, RD and GAK led the design and interpretation. GAK and RP did the statistical analysis. GAK and LD drafted the manuscript. RP, SAC, MA contributed to the interpretation of findings. All authors read and approved the final version of the manuscript.

## Pre-publication history

The pre-publication history for this paper can be accessed here:

http://www.biomedcentral.com/1471-2458/14/64/prepub

## References

[B1] Jaffe HW Valdiserri RO De Cock KM The reemerging HIV/AIDS epidemic in men who have sex with men JAMA 2007 298 20 2412 2414 18042919 10.1001/jama.298.20.2412

[B2] Smith AD Tapsoba P Peshu N Sanders EJ Jaffe HW Men who have sex with men and HIV/AIDS in sub-Saharan Africa Lancet 2009 374 9687 416 422 19616840 10.1016/S0140-6736(09)61118-1

[B3] Beyrer C Baral SD van Griensven F Goodreau SM Chriyaletsak S Wirtz AL Brookmeyer R Global epidemiology of HIV infection in men who have sex with men Lancet 2012 380 9839 367 377 22819660 10.1016/S0140-6736(12)60821-6 PMC3805037

[B4] Beyrer C Sullivan PS Sanchez J Dowdy D Altman D Trapence G Collins C Katabira E Kazatchkine M Sidibe M Mayer KH A call to action for comprehensive HIV services for men who have sex with men Lancet 2012 380 9839 424 438 22819663 10.1016/S0140-6736(12)61022-8 PMC3805059

[B5] National AIDS Control Organization (NACO) Technical report: India HIV estimates New Delhi: National AIDS Control Organization, Ministry of Health & Family Welfare Government of India 2010 Available at. http://naco.gov.in/upload/Surveillance/Reports%20%20Publication/Technical%20Report%20India%20HIV%20Estimates%202010.pdf

[B6] National AIDS Control Organization (NACO) Targeted Interventions Under NACP III, operational guidelines, volume (1), core high risk groups 2007 New Delhi: NACO, Ministry of Health and Family Welfare, Government of India Available at. http://www.naco.gov.in/upload/Publication/NGOs%20and%20targetted%20Intervations/NACP-III.pdf

[B7] National AIDS Control Organization (NACO) Annual Report 2010–11 2011 New Delhi: Department of AIDS Control, Ministry of Health & Family Welfare, Government of India Available at. http://www.naco.gov.in/upload/REPORTS/NACO%20Annual%20Report%202010-11.pdf

[B8] Bill & Melinda Gates Foundation Breaking Through Barriers: Avahan’s Scale-Up of HIV Prevention among High-Risk MSM and Transgenders in India 2010 New Delhi, India: Bill & Melinda Gates Foundation Available at. https://docs.gatesfoundation.org/Documents/breaking-thru-barriers.pdf

[B9] ICMR and FHI360 National Summary Report – India, Integrated Behavioural and Biological Assessment (IBBA), Round 2 (2009–2010) 2011 New Delhi: Indian Council for Medical Research and Family Health International Available at. http://www.nari-icmr.res.in/IBBA/121IBBA_Round_2_NSR.pdf

[B10] Verma RK Collumbien M Homosexual activity among rural Indian men: Implications for HIV interventions AIDS 2004 18 13 1845 1847 15316346 10.1097/00002030-200409030-00014

[B11] Dandona L Dandona R Gutierrez JP Kumar GA McPherson S Bertozzi SM ASCI FPP Study Team Sex behavior of men who have sex with men and risk of HIV in Andhra Pradesh, India AIDS 2005 19 6 611 619 15802980 10.1097/01.aids.0000163938.01188.e4

[B12] Dandona L Dandona R Kumar GA Gutierrez JP McPherson S Bertozzi SM ASCI FPP Study Team How much attention is needed towards men who sell sex to men for HIV prevention in India? BMC Public Health 2006 6 31 16478546 10.1186/1471-2458-6-31 PMC1421390

[B13] Hernandez A Lindan C Mathur M Ekstrand M Madhivanan P Stein ES Gregorich S Kundu S Gogate A Jerajani HR Sexual behavior among men who have sex with women, men, and Hijras in Mumbai, India–multiple sexual risks AIDS Behav 2006 10 Suppl 4 S5 S16 16832600 10.1007/s10461-006-9129-z

[B14] Brahmam GN Kodavalla V Rajkumar H Rachakulla HK Kallam S Myakala SP Paranjape RS Gupte MD Ramakrishnan L Kohli A Ramesh BM IBBA Study Team Sexual practices, HIV and sexually transmitted infections among self-identified men who have sex with men in four high HIV prevalence states of India AIDS 2008 22 Suppl 5 S45 S57 19098479 10.1097/01.aids.0000343763.54831.15

[B15] Phillips AE Lowndes CM Boily MC Garnett GP Gurav K Ramesh BM Anthony J Moses S Alary M Men who have sex with men and women in Bangalore, South India, and potential impact on the HIV epidemic Sex Transm Infect 2010 86 3 187 192 20522632 10.1136/sti.2009.038216

[B16] Schneider JA Saluja GS Oruganti G Dass S Tolentino J Laumann EO Yeldandi V Pitrak D HIV infection dynamics in rural Andhra Pradesh south India: a sexual-network analysis exploratory study AIDS Care 2007 19 9 1171 1176 18058402 10.1080/09540120701336392

[B17] Gutierrez J McPherson S Fakoya A Matheou A Bertozzi SM Community-based prevention leads to an increase in condom use and a reduction in sexually transmitted infections (STIs) among men who have sex with men (MSM) and female sex workers (FSW): the Frontiers Prevention Project (FPP) evaluation results BMC Public Health 2010 10 497 Available at. http://www.biomedcentral.com/1471-2458/10/497 20718977 10.1186/1471-2458-10-497 PMC2940912

[B18] ICMR and FHI360 National Interim Summary Report - India, Integrated Behavioural and Biological Assessment (IBBA), Round 1 (2005–2007) 2007 New Delhi: Indian Council of Medical Research and Family Health International Available at. http://www.nari-icmr.res.in/IBBA/IBBA-NISR.pdf

[B19] ICMR and FHI360 Integrated Behavioral and Biological Assessment: Guidelines for Surveys of Populations at Risk of HIV Infection 2011 New Delhi: Indian Council of Medical Research and Family Health International Available at. http://www.fhi360.org/sites/default/files/media/documents/Integrated%20Behavioral%20and%20Biological%20Assessment%20Guidelines%20for%20Surveys%20of%20Populations%20at%20Risk%20of%20HIV%20Infection.pdf

[B20] Bennett S Woods T Liyanage WM Smith DL A simplified general method for clustersample surveys of health in developing countries World Health Stat Quart 1991 44 3 98 106 1949887

[B21] Kumta S Lurie M Weitzen S Jerajani H Gogate A Row-kavi A Anand V Makadon H Mayer KH Bisexuality, sexual risk taking, and HIV prevalence among men who have sex with men accessing voluntary counseling and testing services in Mumbai India J Acquir Immune Defic Syndr 2010 53 2 227 233 19934765 10.1097/QAI.0b013e3181c354d8 PMC2844633

[B22] Chakrapani V Newman PA Shunmugam M Dubrow R Prevalence and contexts of inconsistent condom use among heterosexual men and women living with HIV in India: implications for prevention AIDS Patient Care STDS 2010 24 1 49 58 20095889 10.1089/apc.2009.0214 PMC2859766

[B23] Joint United Nations Programme on HIV/AIDS (UNAIDS) Making condoms work for HIV prevention: cutting-edge perspectives 2004 Geneva: UNAIDS Available at. http://data.unaids.org/publications/irc-pub06/jc941-cuttingedge_en.pdf

[B24] Ng M Gakidou E Levin-Rector A Khera A Murray CJ Dandona L Assessment of population-level eff ect of Avahan, an HIV-prevention initiative in India Lancet 2011 378 9803 1643 1652 21993161 10.1016/S0140-6736(11)61390-1

[B25] Pickles M Boily MC Vickerman P Lowndes CM Moses S Blanchard JF Deering KN Bradley J Ramesh BM Washington R Adhikary R Mainkar M Paranjape RS Alary M Assessment of the population-level effectiveness of the Avahan HIV-prevention programme in South India: a preplanned, causal-pathway-based modelling analysis Lancet Global Health 2013 1 5 e289 e299 10.1016/S2214-109X(13)70083-4 25104493

